# The Nephroprotective Potential of *Brassica nigra* Sprout Hydroalcoholic Extract against Carbon Tetrachloride-Induced Renal Toxicity in Rats

**DOI:** 10.3390/foods12213906

**Published:** 2023-10-25

**Authors:** Thamer Aljutaily, Sarah M. Almutairi, Hend F. Alharbi

**Affiliations:** Department of Food Science and Human Nutrition, College of Agriculture and Veterinary Medicine, Qassim University, Buraydah 51452, Saudi Arabia; 421200245@qu.edu.sa (S.M.A.); hf.alharbi@qu.edu.sa (H.F.A.)

**Keywords:** *Brassica nigra*, polyphenols, nephroprotection, antioxidative stress, renal toxicity

## Abstract

The nephroprotective potential of the *Brassica nigra* sprout (BNS) hydroalcoholic extract against carbon tetrachloride (CCl_4_)-induced renal toxicity in rats was the object of this study. *B. nigra* sprouts were prepared in the lab to monitor the bio-changes in bioactive compounds during the sprouting for up to 7 days at 17 ± 1 °C and 90% relative humidity. Subsequently, 6-day sprouts of *B. nigra* were selected according to their phenolics and antioxidant activity, extracted, and examined for their nephroprotective and antioxidative stress potential at 250 and 500 mg sprout extracts kg^−1^ bw, *in vivo*. Weight gain, organ weight, lipid profile, atherogenic index, kidney functions, and oxidative stress biomarkers were assessed. The results indicated that the most proficient treatment for weight gain improvement was BNS extract at 500 mg kg^−1^. BNS at 250 mg kg^−1^ was remarked as the lowest weight gain enhancer compared to the NR group. A significant increase in TG, TC, LDL-c, and VLDL-c levels in the rats with CCl_4_-induced renal toxicity, and a significant decrease in HDL level, was noted. The administration of the BNS extract at 250 and 500 mg kg^−1^ considerably attenuated TG, TC, LDL-c, and VLDL-c levels, compared to the NR group. The most efficient treatment for improving the lipid profile was the BNS extract at 500 mg kg^−1^, even better than 250 mg kg^−1^. Administrating the BNS extract substantially attenuated the alterations in the creatinine, urea, and BUN caused by the CCl_4_ injection. The most efficient improvement was markedly recorded with the BNS extract at 500 mg kg^−1^, compared to the NR group. The rats treated with the BNS extract showed significant enhancement in GSH, CAT, and SOD activities and a considerable reduction in MDA levels. Administering the BNS extract at 250 and 500 mg kg^−1^ can efficiently reverse CCl_4_ inhibition of antioxidant enzyme activities, significantly increase GSH, CAT, and SOD, and decrease the MDA levels dose-dependently. The BNS extract at 250 and 500 mg kg^−1^ exhibits nephroprotection and antioxidative stress in a dose-dependent matter. The total nephroprotection % was recorded at 65.18% and 99.21% for rats treated with 250 and 500 mg kg^−1^, respectively. These findings could prove and potentiate the nephroprotective activities of the BNS extract in the range of the given doses. Further clinical studies are highly recommended for confirming the nephroprotection efficiency of the *B. nigra* sprout.

## 1. Introduction

Chronic kidney disease affects 15% of US adults, or 37 million individuals, and it is the ninth leading cause of mortality (CKD) [1]. CKD is most often caused by diabetes, followed by high blood pressure and glomerulonephritis [2]. CCl_4_ generates active free radicals that damage the liver, kidney, lungs, testes, and blood. Its strong hepatotoxin can harm the liver. In high doses, this chemical can harm the CNS (CNS). Death or coma often follow persistent CCl_4_ exposure. The chemical can possibly cause cancer and kidney damage [3]. Practically, CCl_4_ was once a popular cleaner (as a degreasing agent in dry cleaning institutions and other industries, and as a spot remover for clothes, furniture, and carpeting in households). It is used in fire extinguishers and grain fumigants to kill insects [4]. According to Ogeturk et al. [5], this solvent damages kidneys acutely and chronically and causes many disease disorders [6]. Surprisingly, the traditional usage of plant seeds and sprouts has risen in recent years, and several studies have verified their functional and therapeutic values against different diseases [7,8]. Plants produce several secondary metabolites with various pharmacological effects. The most popular vegetables are in the Brassicaceae family including broccoli, cabbage, cauliflower, and Brussels sprouts. The health effects of the Brassica sprouts have been recently reviewed [9]. They are rich in bioactive components such as polyphenols, carotenoids, tocopherols, glucosinolates (GLs), vitamins A and E, minerals, fiber, and low fat [7,8]. New research shows that they supply a lot of antioxidants due to their high phenolic compounds, carotenoids, steroids, tocopherols, and ascorbic acid content [10,11,12,13], in addition to phytosterols, phytoalexins, terpenes, and tocopherols [14,15].

Epidemiological studies suggest these compounds may protect against reactive oxygen species. It relieves pain from the reproductive system and from metabolic illnesses such as diabetes, epilepsy, gastrointestinal disorders, arthritis, bronchial asthma, mouth ulcers, malaria, and depression [16]. It also reduces cancer, osteoporosis, type 2 diabetes, hypertension, and stroke risk [17]. A literature assessment of the sprouts’ biological activity, health benefits, and bioavailability, in vitro and in vivo was made [18,19,20]. Recently, sprouted foods have gained popularity, including sprouts from various seeds, such as alfalfa, buckwheat, red cabbage, and broccoli [21,22]. Sprouts contain biologically active elements with health advantages [23]. Such compounds are known to possess antioxidant activity; it is noted that sprouts are consumed due to their high bioactive component content, antiviral activity, immune stimulant activity, antidiabetic activity [23], anti-hypocholesterolemic and neuroprotective functions [24], antimicrobial, anticancer, anti-obesity properties [22], and immune-boosting agents [25]. For instance, Gawlik-Dziki et al. [26] revealed chemopreventive potential for broccoli sprouts on human stomach cancer. Young et al. [27] demonstrated its efficiency against *Helicobacter pylori* infection; significant attenuation of mucosal malondialdehyde and lipid peroxidation prevention were remarked. Nakamura et al. [28] indicated significant decreases in heart rates and serum triglycerides. The study of Drozdowska et al. [29] confirmed the anti-cancer impact of GLs and isothiocyanates [30]. On the other side, studies have demonstrated that sprout antioxidants protect against oxidative stress and block protoenzymes that convert carbs into simple sugars [31,32,33,34], reduce advanced glycation (AGE) end-product generation [35], and improve fasting blood glucose and insulin resistance [36]. In vitro and in vivo studies have validated sprouted seeds’ significance in heart-related disorders [37], improved cholesterol metabolism and decreased oxidative stress markers [38], lowered chronic inflammation [39], and have anti-atherosclerotic activity [40]. Research summaries on recent sprout advances have been released [41,42,43].

In spite of *B. nigra* seeds and their sprouts having various health benefits, as mentioned above, and as no investigation against nephrotoxicity conditions was carried out, the *B. nigra* sprouts were selected for the current study. The relationship between sprouts and health has received a lot of attention, but there are still some areas that need more study. An updated assessment of active metabolites, technological developments, and sprouted seed applications is required. The evaluation is notably helpful to sprout developers who need constantly updated information to manufacture inexpensive but high-quality seed sprouts for dietary systems. This study focuses on the *B. nigra* sprouts, one of the least studied Brassica plant species. Therefore, the current work examines alterations in antioxidant defense enzymes and the *B. nigra* sprouts’ nephroprotective ability against CCl_4_-induced renal damage in rats.

## 2. Materials and Methods

### 2.1. Sprouting of B. nigra

Seeds of *B. nigra* L. were purchased from True Leaf Market (www.trueleafmarket.com). The cleaned *B. nigra seeds* were sanitized by deeping into a sodium hypochlorite solution (CDH, India) (1%) for 2 min before sprouting. The seeds were evenly distributed on 7 × 35 cm plastic trays after being rinsed 3 times in sterilized distilled water (sd.H_2_O). The seed sprouter was filled with sanitized seeds. The sprouting procedure was performed in an atomizer-equipped temperature-controlled seed sprouter (Easygreen, Biovie Co., Langlade, France) with a relative humidity of 90–94% and 17 ± 1 °C [44]. The selected sprouts with high polyphenols and antioxidant contents (as presented in our previous publication [25]) were gradually dried using the drying program for the cultures for 24 h, after which dried sprouts were ground and extracted for biological evaluation [45]. Approximately 500 g of *B. nigra* sprouts were extracted 3 times with 2500 mL ethanol (50%) to prepare *B. nigra* hydroalcoholic extract, filtered, concentrated by a rotary evaporator at 40°, frozen overnight, then freeze-dried for 96 h at −52 °C using (CHRIST, Alpha 1–2 LD plus, Osterode, Germany) and 0.032 mbar [46]. Freeze-dried samples were powdered to prepare homogeneous powder and then kept in a dark glass bottle at 4 ± 1 °C until used.

### 2.2. Rats Experiment Design

Thirty-two male Wistar rats weighing 180–200 g were obtained from the Experimental Animal Care Center, College of Pharmacy, King Saud University, Riyadh, Saudi Arabia. Rats were housed inside cages at room temperature (25 °C) and humidity (40–45%) with exposure to 12 h of light and 12 h of darkness. Rats were acclimatized for a week before the start of the experiment with feeding of a commercial standard diet (NRC, 1985) and water *ad libitum*. Consequently, the rats’ boxes will be divided into 4 groups (*n* = 8/group). Group 1: Normal rats do not receive any treatment (NR). Group 2: Positive control received CCl_4_ (Sigma-Aldrich, USA) at 1.0 mL kg^−1^ and was fed a commercial standard diet (CCl_4_). Group 3: Positive control received CCl_4_ at a dose of 1.0 mL kg^−1^ and was provided a commercial standard diet (diet + 250 mg *B. nigra* sprouts extract Kg^−1^ body weight) (CCl_4_+BN250). Group 4: positive control received CCl_4_ at a dose of 1.0 mL kg^−1^ and was fed a commercial standard diet (diet + 500 mg *B. nigra* sprouts extract Kg^−1^ body weight) (CCl_4_+BN500).

### 2.3. Weight Gain, Blood and Organs Measurements

The body weights of each rat were measured weekly throughout the experiment. Cumulative weight gain was calculated according to Almundarij et al.’s [47] formula. At the end of the experiment, the experimental rats were anesthetized with diethyl ether, and blood samples were collected from the jugular vein. Immediately after the collection, the blood tubes were submitted to centrifugation (4000 rpm at 10 °C) for 30 min, and the serum obtained was preserved at −18 °C until use. After collecting blood samples, three rats from each group were humanely sacrificed using cervical dislocation under anesthetization. The liver, kidneys, and spleen were then excised carefully and weighed using a sensitive scale. The relative weight was calculated according to the formula:

Relative organs’ weight = (organ’s weight (g)/final body weight (g)) × 100 (1)

### 2.4. Determination of Lipid Profile

Lipid profile, including triglycerides (TG, mg dL^−1^) and total cholesterol (TC, mg dL^−1^) using enzymatic colorimetric test kits applying the GPO-PAP method and CHOD-PAP-method, respectively, according to manufacturer protocols, were determined. High-density lipoproteins (HDL, mg dL^−1^) were determined using an enzymatic colorimetric direct homogenous test kit, following manufacturer protocols. All biochemical examination kits were purchased from Human Co. (Gesellschaft für Biochemica und Diagnostica mbH. Max-Planck-Ring 21. Gesellschaft für Biochemica und Diagnostica mbH, Wiesbaden, Germany). Low-density lipoproteins (LDL, mg dL^−1^) and very-low-density lipoproteins (VLDL, mg dL^−1^) were mathematically calculated according to Friedewald et al. [48]. Then, the total lipid (TLs, mg dL^−1^) in blood serum was calculated according to Bernert et al. [49] using a simplified equation as follows:

TLs = (2.27 × TC) + TG + 62.3(2)

### 2.5. Determination of Atherogenic Index

The atherogenic index (AI), as an essential predictor of atherosclerosis and reflecting the ratio of TG and HDL in blood, was calculated according to Nwagha et al. [50].

### 2.6. Determination of Kidneys’ Functions

Kidneys’ functions, such as creatinine (mg dL^−1^) and urea (mg dL^−1^) levels, using photometric colorimetric and GLDH enzymatic test kits, respectively, were determined according to the manufacturer’s instructions. Blood urea nitrogen (BUN, mg dL^−1^) was calculated by multiplying urea concentration by 0.47. All biochemical examination kits were purchased from Human Co. (Gesellschaft für Biochemica und Diagnostica mbH. Max-Planck-Ring 21. Gesellschaft für Biochemica und Diagnostica mbH, Wiesbaden, Germany).

### 2.7. Oxidative Stress Biomarkers

Reduced glutathione (GSH, µg dL^−1^) using a GSH colorimetric assay kit, Superoxide dismutase (SOD, UL^−1^) activity using SOD typed activity assay kit, and Catalase (CAT, UL^−1^) activity using a CAT activity assay kit were determined following instructions of the manufacturer (Elabscience, Biotechnology Inc., Houston, TX, USA). Lipid peroxidation was assessed using a malondialdehyde (MDA, nmol mL^−1^) colorimetric assay kit by measuring thiobarbituric acid reactive substance (TBARS) and expressed in terms of MDA content according to manufacturer protocol Elabscience, Biotechnology Inc., Houston, TX, USA). All oxidative stress biomarkers were determined using a blood chemistry analyzer (HumaLyzer 4000, HUMAN Gesellschaft für Biochemica und Diagnostica mbH, Wiesbaden, Germany).

### 2.8. Nephroprotection Percentage

The nephroprotection (NP) percentages of groups giving 250 and 500 mg *B. nigra* sprout extracts Kg^−1^ were separately calculated for each biochemical parameter according to Wakchaure et al. [51] using the following equation:

(3) $$\mathrm{NP}\%=[1-\frac{\left(T-N\right)}{\left(P-N\right)}]\times 100$$

where, T = mean value of the treatment group, P = mean value of the positive control group, and N = mean value of the negative control group.

Moreover, the total nephroprotection percentage (TFP %) was compared to the NR group considering that the NR group is 100%, as follows:

(4) $$\mathrm{TNP}\%=\frac{{Average\;of\;NP\%\;of\;each\;biochemical\;parameter}_{T}}{{Average\;of\;NP\%\;of\;each\;biochemical\;parameter}_{N}}\times 100$$

where T = refers to the treatment group and N = refers to the negative group.

### 2.9. Statistical Analysis

The statistical analysis will be carried out using the SPSS program (ver. 19) by applying the analysis of variance (one-way ANOVA) regarding experimental design. Applying a significance level of 0.05 and Tukey’s test was achieved according to Steel [52].

## 3. Results and Discussion

### 3.1. Effect of B. nigra Sprout Extract Administration on Weight Gain % and Organ Relative Weight in Rats with CCl_4_-Induced Renal Toxicity

The weight gain % and organ relative weight % in rats with CCl_4_-induced renal toxicity were examined; the data are tabulated in Table 1. At the end of the experiment, the injection of CCl_4_ increased the rat’s weight. The *B. nigra* sprout extract at 500 mg kg^−1^ was the most effective treatment for considerably restoring the weight of the rats. Compared to the NR group, the lowest weight gain enhancer was the BNS at 250 mg kg^−1^. However, there was a dose-dependent relationship between the dose of the *B. nigra sprout* extract consumed and the rate of weight restoration.

Significant improvements were found in the liver relative weight % after receiving the *B. nigra* sprout extracts at 250 and 500 mg Kg^−1^, especially with 500 mg Kg^−1^ producing the most noticeable results. The formation of fat vacuoles and the production of extrahepatic cholesterol and triglycerides likely contributed to the exponential rise in liver weight, as seen in the CCl_4_ group [53]. Similarly, the rise of hepatic hydroxyproline content in rats with liver fibrosis produced by biliary obstruction led to large increases in liver weight [54]. Previous research using a CCl_4_-induced liver injury model demonstrated that the relative liver weight was a more sensitive indication of hepatotoxicity than the relative liver weight [55].

### 3.2. The Hypolipidemic Efficiency of B. nigra Sprout Extract

The hypolipidemic efficiency of the *B. nigra* sprout extracts at 250 and 500 mg kg^−1^ in rats with CCl_4_-induced renal toxicity was examined; the data are illustrated in Table 2. The rats with CCl_4_-induced renal damage showed a considerable rise in TG, TC, LDL-c, and VLDL-c levels. However, there was a discernible drop in HDL levels (NR) compared to normal rats. Compared to the CCl_4_ group, administering the BNS sprout extracts at 250 and 500 mg kg^−1^ significantly reduced TG, TC, LDL-c, and VLDL-c values. The most efficient treatment for improving the blood profile was the BNS sprout extract at 500 mg kg^−1^, even better than at 250 mg kg^−1^. The TG levels were attenuated by 31.44% and 42.85% when the rats were treated with the BNS sprout extract at 250 and 500 mg Kg^−1^, respectively. Similarly, the TC was decreased by 25.76% and 38.91% when administrating the *B. nigra* sprout extracts at 250 and 500 mg Kg^−1^, respectively.

Interestingly, the rate of the HDL-c rise for the *B. nigra* sprout extracts at 250 and 500 mg Kg^−1^ was 29.17% and 54.17%, respectively. At 250 and 500 mg kg^−1^, the *B. nigra* sprout extract reduced LDL-c by 10.24 and 64.41%, respectively. The VLDL-c level improved in a dose-dependent manner associatively with treatments. The best treatment was the *B. nigra* sprout extract at 500 mg kg^−1^, which lowered VLDL-c by 42.86% compared to the CCl_4_ group. It is important to note that polyphenols’ antioxidant activity is mostly due to their redox characteristics, allowing them to operate as reducing agents, hydrogen donors, singlet oxygen quenchers, and metal chelators [56,57].

In addition, our results demonstrated a significant drop with a reasonable proportion of improvements in triglycerides, cholesterol, and its derivatives after the treatment with the *B. nigra* sprout extract. The consequences of our results can be interpreted, based on the phytochemicals of the BNS sprout extract acting as hyperlipidemic and hypercholesterolemic agents and coinciding with the work of Lee et al. [58] where they examined the effects of *B. juncea* leaf extract on lipid profiles and fat storage. Although further research into the underlying mechanisms is necessary, the current findings imply that the *B. nigra* sprout extract is clinically functional for treating dyslipidemia. Harnafi et al. [59] demonstrated that the phenolic-rich extract from Prunus dulcis hulls improved lipid metabolism and protected against lipoprotein oxidation in mice with hyperlipidemia caused by high-fat diet feeding. Our phytochemical analysis indicated that the *B. nigra* sprout extract is rich in phenolics and flavonoids. These significant hypocholesterolemic and antihyperlipidemic effects and antioxidant activity may be partly due to the *B. nigra* sprout extract’s phenolic constituents, especially caffeic, cinnamic, benzoic, and rosmarinic as phenolic acids, and kaempferol, myricetin, quercetin, rutin, and resveratrol as flavonoids, which were in agreement with Belguith-Hadriche et al. [60].

### 3.3. Effect of B. nigra Sprout Extract on Total Lipids (TLs) Content and Atherogenic Index (AI)

**Figure 1:**
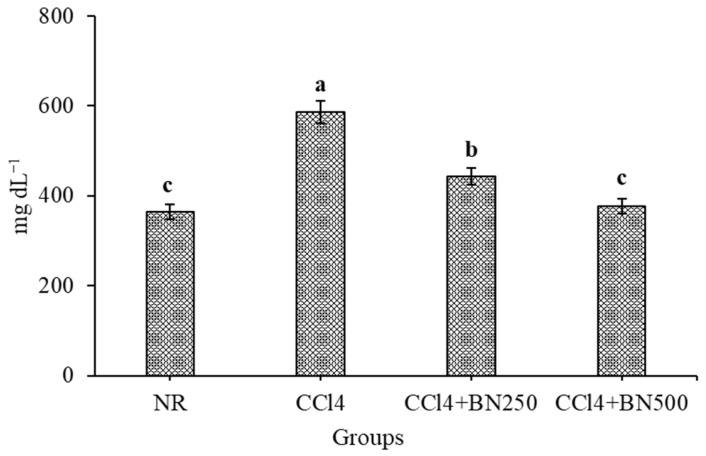
Effect of *B. nigra* sprout extract at different doses on TLs content in the blood of rats with CCl_4_-induced renal toxicity (mean ± SE), *n* = 8, ^a,b,c^: Bars not sharing similar letters differed significantly (*p* > 0.05), For experimental groups, see Materials and Methods.

**Figure 2:**
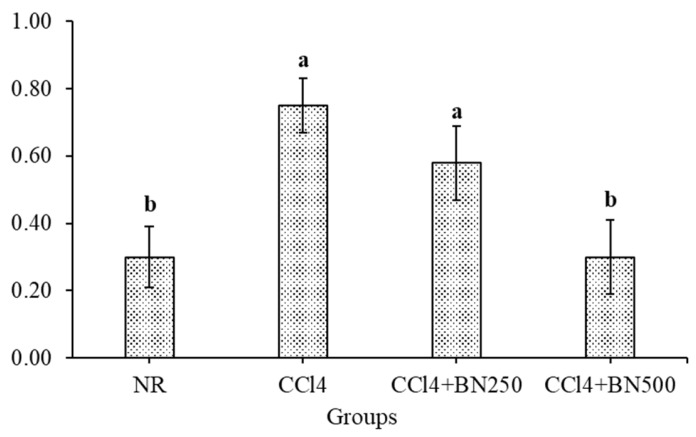
Effect of *B. nigra* sprout extract at different doses on AI in rats with CCl_4_-induced renal toxicity (mean ± SE), *n* = 8, ^a,b^: Bars not sharing similar letters differed significantly (*p* > 0.05).

The efficiency of the *B. nigra* sprout extracts at 250 and 500 mg kg^−1^ on TLs in the rats with CCl_4_-induced renal toxicity was calculated; the data are illustrated in Figure 1. Interestingly, the TLs were significantly increased after the CCl_4_ injection (G2) compared with normal rats (NR). The administration of the *B. nigra* at sprout extracts at 250 and 500 mg kg^−1^ significantly reduced the TLs content in a dose-dependent matter. The reduction rate was 24.53% and 35.82% after giving 250 and 500 mg kg^−1^, respectively. Interestingly, Lee et al. [58] indicated, based on the phytochemicals of the BNS extract, it exhibited hyperlipidemic and hypercholesterolemic activity on fat deposition and lipid profiles. Thus, TLs could be improved as the *B. nigra* sprout extract is clinically functional for treating dyslipidemia. These findings were in agreement with our obtained results. Also, Harnafi et al. [59] indicated that phenolic-rich extracts are capable of improving the lipid metabolism in obese rats and also prevent lipoproteins’ oxidation, in vivo. Subsequently, the efficiency of the *B. nigra* sprout extracts at 250 and 500 mg kg^−1^ on the AI in the rats with CCl_4_-induced renal toxicity was calculated; the data are illustrated in Figure 2. Interestingly, the AI was significantly increased after the CCl_4_ injection (G2) when compared with normal rats (NR). The most efficient treatment for attenuating the atherogenicity complication was the BNS at 500 mg kg^−1^ (G4), which presented a better effect than the BS at 250 mg kg^−1^ (G3).

As Lee et al. [58] indicated, based on the phytochemicals of BNS extract that exhibited hyperlipidemic and hypercholesterolemic activity on fat deposition and lipid profiles, the AI could be improved as the *B. nigra* sprout extract is clinically functional for treating dyslipidemia. The administration of the plant-based materials reduced the concentrations of induced serum inflammatory biomarkers, and its protective effect against cardiovascular risk might be due to its polyphenol content [61]. These findings were in agreement with our obtained results.

### 3.4. Effect of B. nigra Sprout Extracts on Kidneys’ Functions in Rats with CCl_4_-Induced Renal Toxicity

The nephroprotective efficiency of the *B. nigra* sprout extracts at 250 and 500 mg kg^−1^ on the kidneys’ functions in the rats with CCl_4_-induced renal toxicity is illustrated in Table 3. Serum concentrations of creatinine, urea, and blood urea nitrogen (BUN) were significantly higher in the CCl_4_-injected rats than in the NR-injected rats [47]. The creatinine, urea, and BUN levels elevations after the CCl_4_ injection were significantly reduced by the BNS sprout extract at 500 mg kg^−1^. The creatinine, urea, and BUN levels were attenuated after the administration of the BNS sprout extracts at 250 and 500 mg kg^−1^ in a dose-dependent manner. Comparing the lower dose to the higher dose, it could be concluded that creatinine attenuated by 14.15% and 33.02%, respectively.

A similar trend was observed in the urea level, whereas 36.84 and 46.56% were recorded for the *B. nigra* sprout extracts at 250 and 500 mg kg^−1^, respectively. A higher efficiency was noticed for the BNS sprout extract at 500 mg kg^−1^ on BUN, with about 50% improvement. The CCl_4_-induced nephrotoxicity has been used as a model system to evaluate the nephroprotective potential of various plant extracts and medicines in various research [62,63]. The purpose of this research was to examine the nephroprotective and antioxidant properties of the *B. nigra* sprout extracts in rats subjected to CCl_4_-induced kidney injury. The present investigation found that creatinine, urea, and BUN levels were considerably higher in the CCl_4_ treatment group compared to the NR group. This may be due to CCl_4_ intoxication causing free radical generation in several organs, such as the liver, kidneys, lungs, brain, and blood [64].

Another thing that has been noticed after injecting the rats with CCl_4_ is that the chemical is spread out more uniformly in their kidneys than in their livers [65]; since cytochrome P450 is mostly located in the cortex of the kidney, it has a high affinity for CCl_4_. Free radicals such as trichloromethyl (CCl_3_^•^) and trichloromethyl peroxyl (CCl_3_O_2_^•^) are produced in large quantities when CCl^4^ is broken down [66]. These free radicals cause cell death by reacting with an intracellular protein, lipids in the cell membrane, and DNA, resulting in denaturation of the protein, peroxidation of the lipids, and oxidative damage to the DNA [67].

### 3.5. Effect of B. nigra Sprout Extracts on Antioxidant Biomarkers in Rats with CCl_4_-Induced Renal Toxicity

Injecting CCl_4_ as an oxidant agent and oxidative stress enhancer dramatically decreases GSH, CAT, and SOD enzyme levels, as indicated in Table 4. The rats given CCl_4_ had a higher concentration of MDA in their blood serum than the NR group. The activity of the antioxidant enzymes GSH, CAT, and SOD, as well as a significant decrease in the MDA levels, were significantly enhanced in rats treated with the *B. nigra* sprout extract. The sprout extract from *B. nigra* increased GSH by 50.36 and 109.66% at doses of 250 and 500 mg kg^−1^, respectively. The better antioxidant attenuation combats the autoxidation process, resulting in MDA reductions of 28.98 and 39.78% in the B. nigra sprout extracts at 250 and 500 mg kg^−1^, respectively. These results suggest that administering both the *B. nigra* sprout extracts at 250 and 500 mg kg^−1^ can reverse CCl_4_ inhibition of antioxidant enzyme activities, significantly increase GSH, CAT, and SOD, and decrease MDA levels dose-dependently. These observations indicate that the *B. nigra* sprout extracts at 250 and 500 mg kg^−1^ exhibits a potential antioxidant activity against CCl_3_• and CCl_3_O_2_• as free radicals from CCl_4_ [66]. In the same context, accelerating antioxidant activity in rats were 2.56% and 63.10% in CAT levels and 36.48 and 69.73% in SOD levels when the rats were administrated the *B. nigra* sprout extracts at 250 and 500 mg kg^−1^, respectively. Our result is in agreement with Rajamurugan et al. [68], who indicated that crude methanol extract of *B. nigra* exhibits antioxidative stress and accelerates the oxidation enzymes, and scavenges the free radicals in biological systems. This may be due to its superior content of phytochemicals and bioactive substances [16,69].

The oxidative damage induced by CCl_4_ toxicity can be prevented, at least in part, by using plant extracts with antioxidant activity [70]. The results of the current investigation corroborated with this finding and showed that the GSH, CAT, and SOD levels were all raised after consuming the *B. nigra* sprout extract. Our findings may be in full agreement with Keshari et al. [71] since it was found that stress-induced production of antioxidant defense enzymes and intracellular GSH modification required the activation of the Nrf2/ARE pathway [72]. GSH has many biological roles including protection against reactive oxygen and nitrogen species. For instance, it (i) is a reserve form of cysteine; (ii) stores and transports nitric oxide; (iii) participates in the metabolism of estrogens, leukotrienes, and prostaglandins, and it is involved in the reduction of ribonucleotides to deoxyribonucleotides and the maturation of iron-sulfur clusters of diverse proteins; (iv) operates certain transcription factors (particularly those involved in the redox signaling pathway); and (v) detoxifies many endogenous compounds and xenobiotics [73]. It has a function in cell differentiation, proliferation, and apoptosis; hence, GSH homeostasis disruptions are linked to the etiology and/or progression of many human diseases, including cancer, aging, cystic fibrosis, and cardiovascular, inflammatory, immunological, metabolic, and neurological diseases [74].

### 3.6. The Nephroprotection Percentage of B. nigra Sprout

The nephroprotection percentage (relative to the negative control (NR) and positive (CCl_4_) groups) of kidney functions such as creatinine, urea, and BUN data are illustrated in Table 5. The response of nephroprotection was presented in a dose-dependent matter, as seen above. The total nephroprotection percentage was recorded at 65.18% and 99.21% for the rats treated with 250 and 500 mg kg^−1^, respectively. These findings could prove and potentiate the nephroprotection efficiency of the *B. nigra* sprout extract in the range of given doses. This may be due to the presented and developed phenolics and antioxidant substances in *B. nigra* sprouts, which may be related to improving the antioxidant capacity and combating nephrotoxicity [47,63,75]. They demonstrated that numerous medicinal herbs reduce the biochemical, functional, and structural kidney toxicity of numerous medicines and poisons, representing effective nephroprotective alternatives. Several mechanisms proved that plant-based extracts exhibited nephroprotection by enhancing antioxidant enzymes, inhibiting inflammatory cytokine production, and decreasing apoptosis and necrosis.

## 4. Conclusions

Nephroprotective properties of the BNS hydroalcoholic extract against CCl_4_-induced kidney injury in rats were investigated. To study the bio-changes in bioactive chemicals in *B. nigra* sprouts, *B. nigra* seeds were sprouted under lab conditions for up to 7 days at 17 °C and 90% relative humidity. After selecting for high levels of phenolics and antioxidant activity, the sprouts of *B. nigra* were tested in vivo at doses of 250 and 500 mg BNS extract kg^−1^ bw for their nephroprotective and antioxidative stress potential. A substantial attenuation effect in TG, TC, LDL-c, and VLDL-c levels compared to the NR group has been remarked in a dose-dependent manner. The creatinine, urea, and BUN changes from the CCl_4_ injection were significantly reduced by the *B. nigra* sprout extract. The *B. nigra* sprout extracts at 250 and 500 mg kg^−1^ protects kidneys and reduces oxidative stress. Interestingly, rats given an extract of *B. nigra* sprouts showed improved levels of GSH, CAT, and SOD activity, as well as reduced levels of MDA. A dose-dependent increase in GSH, CAT, and SOD, as well as a decrease in MDA levels, remarked considerable changes in the oxidative system of treated rats. The rats given 250 and 500 mg kg^−1^ showed 65.18 and 99.21% nephroprotection, respectively. In conclusion, these findings could prove and potentiate the nephroprotection efficiency of the *B. nigra* sprout extract in the range of given doses. Further clinical studies are highly recommended for confirming the nephroprotection efficiency of the *B. nigra* sprout.

## Figures and Tables

**Table 1:** Weight gain % and organ weight of CCl_4_-induced renal toxicity in rats fed different doses of *B. nigra* sprouts extract (mean ± SE), *n* = 8.

Items	Groups			
	NR	CCl_4_	CCl_4_ + BN250	CCl_4_ + BN500
BW gain %	16.94 ^b^ ±1.91	22.18 ^a^ ±4.83	14.68 ^b^ ±1.49	16.74 ^b^ ±4.15
Relative Liver weight %	3.29 ^b^ ±0.18	3.79 ^a^ ±0.26	3.15 ^b^ ±0.09	3.13 ^b^ ±0.11
Relative Kidney’s weight %	0.66 ^b^ ±0.01	0.68 ^b^ ±0.03	0.73 ^a^ ±0.01	0.67 ^b^ ±0.02
Relative Spleen weight %	0.21 ^ab^ ±0.02	0.22 ^a^ ±0.01	0.21 ^a^ ±0.00	0.18 ^b^ ±0.01

SE: Standard error, BW: Body weight, ^a,b^: No significant difference (*p* > 0.05) between any two means within the same row with similar superscripted letters.

**Table 2:** Effect of *B. nigra* sprouts extract at different doses on the lipid profile of rats with CCl_4_-induced renal toxicity (mean ± SE), *n* = 8.

Groups	Lipid Profile Parameters (mg dL^−1^)				
	TG	TC	HDL-C	LDL-C	VLDL-C
NR	80.74 ±3.25 ^c^	97.40 ±6.76 ^bc^	44.38 ±7.04 ^a^	36.88 ±10.31 ^c^	16.15 ±0.65 ^c^
CCl_4_	155.04 ±6.85 ^a^	162.75 ±12.28 ^a^	30.00 ±4.91 ^bc^	101.75 ±15.79 ^a^	31.01 ±1.37 ^a^
CCl_4_ + BN250	106.30 ±5.49 ^b^	120.82 ±6.91 ^b^	38.75 ±12.95 ^b^	60.81 ±8.82 ^b^	21.26 ±1.10 ^b^
CCl_4_ + BN500	88.60 ±5.78 ^c^	99.42 ±5.30 ^bc^	46.25 ±4.98 ^a^	36.69 ±4.80 ^c^	17.72 ±1.16 ^c^

TG: Triglycerides, TC: Total cholesterols, HDL-C: High-density lipoprotein-cholesterols, LDL-C: Low-density lipoprotein-cholesterols, and VLDL-C: Very low-density lipoprotein-cholesterols, ^a,b,c^: There is no significant difference (*p* > 0.05) between any two means within the same column with the same superscripted letters.

**Table 3:** Effect of *B. nigra* sprouts extract at different doses on Kidneys’ functions in rats with CCl_4_-induced renal toxicity (mean ± SE), *n* = 8.

Groups *	Kidneys’ Functions		
	Creatinine (mg dL^−1^)	Urea (mg dL^−1^)	BUN (mg dL^−1^)
NR	0.73 ± 0.02 ^c^	37.76 ± 7.10 ^b^	17.75 ± 3.34 ^b^
CCl_4_	1.06 ± 0.02 ^a^	73.73 ± 11.03 ^a^	34.66 ± 5.19 ^a^
CCl_4_ + BN250	0.91 ± 0.05 ^b^	46.57 ± 2.47 ^b^	21.89 ± 1.16 ^b^
CCl_4_ + BN500	0.71 ± 0.01 ^c^	39.40 ± 4.17 ^b^	18.51 ± 1.96 ^b^

*: For experimental groups, see Materials and Methods, ^a,b,c^: No significant difference (*p* > 0.05) between any two means within the same column with the same superscripted letters.

**Table 4:** Effect of *B. nigra* sprout extract at different doses on antioxidant biomarkers in rats with CCl_4_-induced renal toxicity (mean ± SE), *n* = 8.

Groups	Antioxidant Biomarkers			
	GSH (µg dL^−1^)	MDA (µ mol mL^−1^)	CAT (U L^−1^)	SOD (U L^−1^)
NR	239.82 ± 18.94 ^b^	24.14 ± 0.82 ^c^	265.59 ± 9.75 ^b^	137.65 ± 5.00 ^a^
CCl_4_	158.03 ± 9.73 ^c^	39.72 ± 0.72 ^a^	229.33 ± 17.32 ^b^	77.57 ± 2.95 ^c^
CCl_4_ + BN250	237.61 ± 31.75 ^b^	28.21 ± 1.02 ^b^	235.21 ± 6.23 ^b^	105.87 ± 4.78 ^b^
CCl_4_ + BN500	331.33 ± 19.07 ^a^	23.92 ± 0.51 ^c^	374.04 ± 51.02 ^a^	131.66 ± 3.39 ^a^

GSH: Reduced glutathione, CAT: Catalase, SOD: Superoxide dismutase, MDA: Malonaldehyde, ^a,b,c^: No significant difference (*p* > 0.05) between any two means within the same column with similar superscripted letters.

**Table 5:** Nephroprotection percentage of *B. nigra* sprouts extract at different doses on kidney functions in rats with CCl_4_-induced renal toxicity.

Groups *	Groups *		
	NR	CCl_4_ + BN250	CCl_4_ + BN500
Creatinine	100	44.50	106.70
Urea	100	75.52	95.46
BUN	100	75.52	95.46
TNP%	100	65.18	99.21

*: For experimental groups, see Materials and Methods, TNP%: Total nephroprotection %.

## Data Availability

Data is contained within the article.
